# 

**Published:** 1869-09-15

**Authors:** 


VOL. XXVI. NUMBERS 17 AND 18.

THE

### CHICAGO

### Medical Journal.

PUBLISHED SEMI-MONTHLY.

J. ADAMS ALLEN, M. D., LL. D.,

WALTER HAY, A. M., M. D.,

*EDITORS.*

**September 1st and 15th.**

1869.

J. WOLCOTT HOOKER, 12 Union Building,

CHICAGO.

HORTON & LEONARD, PRINTERS, 104 AND 106 RANDOLPH STREET.

